# Two rare cases of myelin oligodendrocyte glycoprotein antibody-associated disorder in children with leukodystrophy-like imaging findings

**DOI:** 10.1186/s12883-023-03294-4

**Published:** 2023-06-27

**Authors:** Xin Wang, Ruibin Zhao, Huafang Yang, Chong Liu, Qing Zhao

**Affiliations:** 1 Second Department of Neurology, Hebei Children’s Hospital, Shijiazhuang, China; 2 School of Medical Imaging, Hebei Medical University, Shijiazhuang, China

**Keywords:** Leukodystrophy-like, Myelin oligodendrocyte glycoprotein antibody-associated disorder, Acquired demyelinating syndromes, Brain magnetic resonance imaging, Children

## Abstract

**Background:**

Children with acquired demyelinating syndromes (ADS) whose sera are positive for myelin oligodendrocyte glycoprotein (MOG) immunoglobulin (IgG) can be diagnosed with MOG-IgG associated disorder (MOGAD). Cases with leukodystrophy-like imaging findings with recurrent MOGAD have rarely been reported.

**Case presentation:**

Two children with MOGAD, whose onset age was 6 months and 3 years, respectively, were admitted to the hospital due to fever and altered consciousness. In both children, MOG-IgG was detected in the serum using live cell-based assay. Brain magnetic resonance imaging (MRI) revealed leukodystrophy-like lesions with diffuse bilateral white matter. Cerebrospinal fluid (CSF) analysis showed mild pleocytosis with normal or slightly increased protein levels and no oligoclonal bands. Metabolic and inflammatory blood/CSF markers were all negative. Full exon gene testing revealed normal results, and nuclear and mitochondrial DNA were normal. Despite regular immunotherapy and reduction of lesions based on brain MRI results, the patients repeatedly relapsed and had residual neurological dysfunction at 3–4 years of follow-up.

**Conclusions:**

Although MOGAD is a monophasic and benign condition, certain MOGAD patients can experience multiple relapses and residual neurologic deficits. The spectrum of clinical manifestations in MOGAD is wider in children than in previously reported cases, including cases with leukodystrophy-like imaging findings. Such imaging findings along with MOG-IgG may occur recurrently and result in severe neurological prognosis. Patients with extensive and confluent white matter lesions should undergo early testing of MOG-IgG to ensure early therapy. In refractory cases, MOGAD treatment may need to be escalated beyond the current therapy, which means second-line immunotherapy should be performed as early as possible and hormone levels should not be rapidly reduced. Early diagnosis and appropriate treatment may improve the prognosis of children with MOGAD.

## Background

Myelin oligodendrocyte glycoprotein (MOG) is a glycoprotein located on the myelin sheath of oligodendrocytes with antibodies against MOG, which are associated with central nervous system (CNS) demyelination. In children, acquired demyelinating syndromes (ADS) are frequently associated with MOG-IgG, which can be classified as MOG-IgG-associated disorders (MOGAD) [1, 2]. Its heterogeneous clinical manifestations include acute disseminated encephalomyelitis (ADEM), optic neuritis, aquaporin-4-negative neuromyelitis optica spectrum disorders, transverse myelitis, cortical encephalitis, motor deficits, seizures, and cerebellar symptoms. MOGAD can have monophasic or relapsing courses. The disease spectrum may vary from a benign steroid-responsive illness to a disease with a fulminant course with significant disability [3]. Thus, it is important to identify these varying clinical syndromes based on diagnostic, therapeutic, and prognostic implications.

Herein, we describe two cases of MOGAD in children with extensive leukodystrophy-like lesions detected via brain magnetic resonance imaging (MRI), which is rarely reported in the disease. Recurrent attacks can lead to neurological dysfunction.

## Case presentation

### Patient 1

**Fig. 1:**
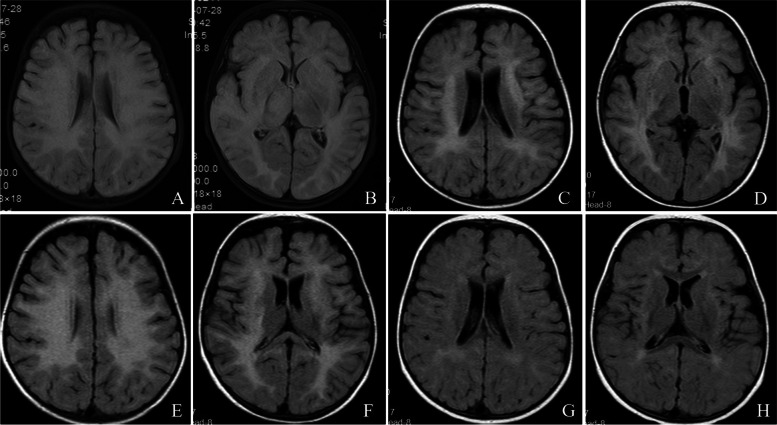
Brain MRI evolution of patient 1 at onset and during follow-up. Images of extensive and confluent bilateral white matter involvement and basic symmetry of lesions for the first episode (**A**, **B**). Bilateral white matter lesions were reduced and new lesions were found in the right occipital cortex (**C**, **D**). Bilateral cerebral hemispheres and centrum semiovale lesions increased (**E**, **F**). Although image lesions were reduced and stabilized after immunotherapy (**G**, **H**), the child showed cognitive impairment and epilepsy

A 6-month-old boy without any family and personal medical history was admitted to the hospital with the first episode of fever and drowsiness for 5 days. Cerebrospinal fluid (CSF) analysis revealed pleocytosis (49 × 10^6^ cells/L with 85% lymphocytes), normal protein level (0.18 g/L), and no oligoclonal bands. Brain MRI showed an extensive and confluent bilateral white matter involvement (Fig. 1A and B). MRI of the orbit and spinal cord showed no lesions. Video electroencephalogram (EEG) showed normal activity. Blood and CSF tests for anti-N-methyl-D-aspartate receptor (NMDAR), AMPA subtype glutamate receptor (AMPAR), LgI1, anti-Hu, Yo, Ri, gamma-aminobutyric acid receptor (GABA-Ab), glutamic acid decarboxylase (GAD65), and anti-aquaporin 4 (AQP4-Ab) showed normal results. Cell-based assay revealed a positive result for MOG-IgG (titer level of 1:32 in the serum). Full exon gene testing revealed normal results, and nuclear and mitochondrial DNA were normal. Intravenous (IV) methylprednisolone and oral prednisone (dosages shown in Table 1) were tapered gradually until discontinuation in 10 months. The patient fully recovered and the brain MRI lesions were reduced.

**Table 1:** The clinical features of the two cases

No	Age (years)	Sex	Episodes	Chief complaint	Brian MRI	VEEG	CSF Cell count	CSF protein	MOG-IgG	Treatments	Follow-up
1	0.5	boy	1	Fever and drowsiness	Symmetry lesions of extensive and confluent bilateral white matter	Normal	49 × 10^6^/L	Normal	1:32	IV methylprednisolone (20 mg/kg for 3 days, halved every 3 days) and oral prednisone tapering gradually until stop taking in ten months	Fully recovered
	1.5		2	Seizure	Bilateral white matter lesions were reduced and new lesions were found in the right occipital cortex	Spiky waves and spiky slow waves during both waking and sleeping periods and one focal attack were detected	Normal	Normal	1:100	IV methylprednisolone (20 mg/kg*3d, halved every 3 days) and gamma globulin (1 g/kg*2d), and oral prednisone tapering until 1.5 mg/kg daily	Epilepsy
	2.3		3	Seizure, unresponsive and language barriers	Extensive leukodystrophy-like lesions, and the lesions were reduced during follow-up	Diffuse slow waves and spinous slow waves	Normal	Normal	1:32	Rituximab (375 mg/m^2^ on Days 1, 7, 15, and 22) was treated every 6 months, and oxcarbazepine and levetiracetam simultaneously	Epilepsy, cognitive degradation
2	3.0	boy	1	Fever, alteration of consciousness	Multiple white matter, basal ganglia and cortex affected (ADEM)	Diffuse slow waves(2-4 Hz)	67 × 10^6^/L	0.62 g/L	1:32	IV methylprednisolone (20 mg/kg for 3 days, halved every 3 days) and oral prednisone tapering until 1.5 mg/kg daily	Fully recovered
	3.7		2	Ataxia	Bilateral cerebellar hemispheres suffered, and multiple lessions were absorbed compared with last time	Normal	Normal	Normal	1:32	IV methylprednisolone (20 mg/kg for 5 days, halved every 3 days) and oral prednisone tapering until 2.0 mg/kg daily	Slight claudication
	4.2		3	Epileptic status and poor memory	Leukodystrophy-like lesions, and the lesions were reduced during follow-up	Diffuse slow waves, spinous slow and cusp slow waves	Not done	Not done	1:32	Rituximab (375 mg/m^2^ on Days 1, 7, 15, and 22) was treated every 6 months, and levetiracetam was added orally	Cognitive impairment, dyspraxia and epilepsy

One year after the first episode, the patient had seizures, showing involuntary movements of his left leg and arm. Furthermore, video EEG showed spiky waves and spiky slow waves during both waking and sleeping periods, and one focal attack was detected while sleeping. Brain MRI revealed reduced bilateral white matter lesions, but new lesions were found in the right occipital cortex (Fig. 1C and D). CSF analysis revealed normal cell counts and protein levels. No autoimmune antibodies for NMDAR, AMPAR, LgI1, anti-Hu, Yo, Ri, GABA-Ab, GAD65, and AQP4-Ab were detected in the serum and CSF, and there was no evidence of viral or bacterial meningoencephalitis. The serum MOG-IgG titer level was 1:100. After treatment with IV methylprednisolone and gamma globulin (dosages shown in Table 1) and tapering of oral prednisone to a dosage of 1.5 mg/kg daily, the patient’s symptoms improved but slight claudication was observed.

At 21 months after the onset, the patient had a recurrence of seizures and cognitive deterioration, presenting with unresponsiveness and language difficulties. Brain MRI still showed extensive leukodystrophy-like lesions, which were larger than those observed the last time (Fig. 1E and F). The serum MOG-IgG titer was 1:32. Autoimmune antibodies in the serum and CSF (NMDAR, AMPAR, LgI1, CASPR2, DPPX, MBP, anti-Hu, Yo, Ri, CV2, GABAB-R-Ab, GAD65, and PNMA2, AQP4-Ab) were negative. CSF analysis showed normal cell counts and protein levels. Video EEG showed diffuse slow waves and spinous slow waves. The patient continued to have seizures despite the administration of oxcarbazepine, levetiracetam, and prednisolone (1.5 mg/kg day). Rituximab (375 mg/m^2^ on days 1, 7, 15, and 22) was administered every 6 months. The patient’s condition stabilized based on the results of his brain MRI (Fig. 1G and H). However, he showed cognitive impairment and epilepsy during the 3-year follow-up. The Wechsler Intelligence Scale for Children (WISC) revealed a total intelligence quotient (IQ) of 68 (Table 1).

### Patient 2

**Fig. 2:**
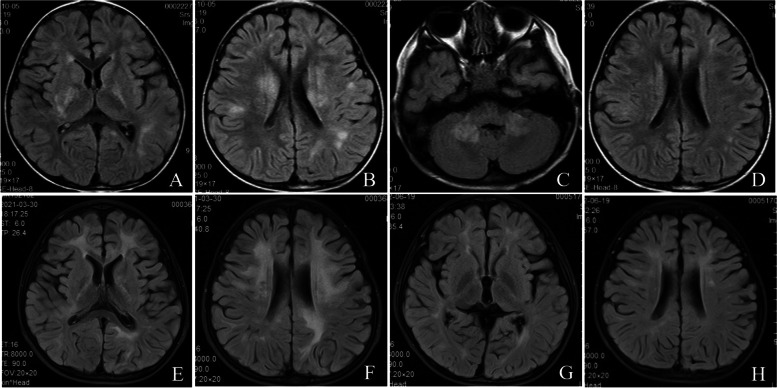
Brain MRI evolution of patient 2 at onset and during follow-up. ADEM-like expressions were observed at onset (A, B). Brain MRI shows scattered cerebellar hemispheres for the second episode (C, D). Leukodystrophy-like lesions at the third attack (E, F) and stabilized with brain atrophy at last follow-up (G, H)

A 3-year-old boy with unremarkable medical history presented with the first episode of typical ADEM, including fever, altered consciousness, and abnormal brain MRI findings, affecting the white matter, posterior limbs of the internal capsules, and cortex (Fig. 2A and B). MRI of the orbit and spinal cord showed no lesions. EEG showed diffuse slow waves (2–4 Hz). CSF analysis revealed pleocytosis (62 × 10^6^ cells/L with 80% of lymphocytes) and an increased protein level (0.62 g/L). Inflammatory antibodies of the serum and CSF, such as NMDAR, AMPAR, LgI1, CASPR2, DPPX, MBP, anti-Hu, Yo, Ri, CV2, GABAB-R-Ab, GAD65, PNMA2, and AQP4-Ab were negative. Moreover, no IgM antibodies were detected for Epstein-Barr virus, human immunodeficiency virus, or hepatitis B. Polymerase chain reaction tests for viral infections, including adenovirus, cytomegalovirus, human herpesvirus 6, enterovirus, and varicella zoster showed negative results. MOG-IgG was later found to be positive in the serum (1:32) and CSF (1:10). The child recovered well after IV methylprednisolone treatment (dosage shown in Table 1) and oral prednisone tapering to 1.5 mg/kg daily.

About 8 months after the onset, the boy suffered a second episode, which manifested as ataxia. Brain MRI showed that the bilateral cerebellar hemispheres were scattered with high signals on FLAIR images, and the white matter and cortex lesions were reduced, compared with the previous MRI (Fig. 2C and D). EEG and CSF analysis showed normal findings. The serum MOG-IgG titer was 1:32. Other inflammatory antibodies in the serum and CSF were negative. After treatment with IV methylprednisolone and gamma globulin (dosages shown in Table 1) and oral prednisone tapered to 2.0 mg/kg daily, the patient's symptoms improved, but he had slight claudication.

At 6 months after the second episode, the patient had a third attack, which presented with status epilepticus and poor memory. Brain MRI showed leukodystrophy-like lesions (Fig. 2E and F). The oral dose of prednisolone was 1.5 mg/kg daily. The MOG-IgG titer level was 1:32 (serum) and 1:10 (CSF). Metabolic screening, full exon gene testing, and nuclear and mitochondrial DNA were negative. Video EEG showed diffuse slow waves, spinous slow waves, and cusp slow waves. Rituximab (375 mg/m^2^ on days 1, 7, 15, and 22) was administered every 6 months. Levetiracetam was added orally simultaneously. The neurological deterioration prompted the monthly use of gamma globulin for 2 months, which led to clinical stability, but no improvement was observed in cognitive function. Moreover, brain MRI indicated atrophic change (Fig. 2G and H). During the 4-year follow-up, the patient showed cognitive impairment, dyspraxia, and epilepsy. WISC showed a total IQ of 62 (Table 1).

## Discussion and conclusions

MOGAD has been shown to be an independent and clinically distinct entity from other ADS. Based on the ages at onset, imaging manifestations, and therapeutic responses, more clinical manifestations of MOGAD have been recognized. It is important to identify these factors for the treatment and prognosis of patients with MOGAD.

In our cases, unusual brain MRIs were reported in two pediatric patients with MOGAD. One of them was only 6 months old, in whom the lesion was not easily distinguished from metabolic or genetic leukodystrophies. The children experienced a progressive-relapsing course of diseases associated with a leukodystrophy-like brain MRI pattern. Although both children responded well to steroid or gamma globulin therapy, and repeat first-line immunotherapies and long-term oral maintenance therapy (6–12 months) were used, they still had three episodes until the second-line immunotherapies were administered. These children showed cognitive impairment, dyspraxia, and seizures during the follow-up period. In both patients, the brain MRI expression of bilateral and confluent white matter changes made differential diagnosis of leukodystrophy difficult. Moreover, serum MOG-IgG positivity in the early disease stage and MRI changes after steroid therapy supported the diagnosis of neurological demyelinating syndrome. Notably, brain MRI of leukodystrophy-like lesions in children with MOGAD may indicate a poor prognosis.

To date, few rare cases of leukodystrophy-like patterns in MOGAD have been reported among children. Netravathi et al.[4] reported 5 cases with leukodystrophy-like MRI manifestations in 93 MOGAD, which were initially considered to be genetically related diseases, and the age onset of the patients was < 5 years, however, the patients experienced repeated attacks, with MOG-IgG finally showing positivity. Yazbeck et al.[5] also reported the cases of two children with relapsing clinical courses who were positive for MOG-IgG; their MRI changes exhibited extensive white matter and gray matter involvement and subsequent brain biopsy revealed cortical inflammatory infiltrates. Clinical and radiological heterogeneities in MOGAD phenotypes are age dependent, which present preferentially with extensive brain involvement in younger children [6]. Our case of MOG-IgG in a 6-month-old child with leukodystrophy-like patterns is a rare case and is consistent with a previous report showing more progressive and multifocal white matter abnormalities in earlier-age onset patients than in later-age onset patients [7]. Thus, the leukodystrophy-like MRI finding of MOGAD in young children may reflect the susceptibility of the myelinating brain.

Seizures are relatively common and serious presentations in MOGAD. The risk of seizures in MOGAD may be associated with sex, age, and most importantly with clinical phenotypes [8]. Seizures are more common in pediatric patients and in MOGAD patients with the cerebral cortical encephalitis phenotype [9]. The mainstay of treatment for seizures secondary to MOGAD consists of immunotherapy along with antiseizure medications. Pediatric MOGAD patients with cerebral monofocal or polyfocal deficits or relapses have a higher probability of developing chronic epilepsy [10]. It is reported that chronic epilepsy may also be accompanied by cognitive changes [11]. The two presented cases of MOGAD with recurrent seizures and neurological aftereffects are consistent with those reported previously. A focal onset seizure is the most common seizure type, with most patients having focal to bilateral tonic–clonic seizures (71.4%), and most focal onset seizures occur in patients with cortical lesions on MRI or with normal radiographic findings. It has also been reported that optic nerve and spinal cord involvement were less likely to occur in MOGAD cases with seizures [12], which is consistent with our findings.

MOG is a highly conserved protein expressed on the surface of oligodendrocytes, which protects its structural integrity and stability. It accounts for only 0.05% of the myelin sheath. MOG-IgG can activate the complement cascade, leading to oligodendrocyte degradation and demyelination [13]. Many genes with reduced expression in oligodendrocytes are involved in myelin lipid synthesis. Oligodendrocyte gap junction (GJ) coupling is a widespread and essential feature of the CNS and is mediated by connexin47 (Cx47) and Cx32. Loss-of-function mutations affecting Cx47 results in severe leukodystrophy [14]. In laboratory studies, MOG expression occurred after the initiation of myelination from birth in mice [15]. Leukodystrophy can be reproduced in mice that lack Cx47, and immunostaining shows activated oligodendrocytes and astrocytes as well as T and B cells [16]. The younger brain, which has uncompacted myelin, may be more susceptible to decreased MOG expression, resulting in immune-mediated injury. We speculate that the expression reduces as myelination continues and is much lower in older children than in younger children. This could explain why leukodystrophy-like is more common in younger children. Furthermore, it should be determined whether leukodystrophy-like demyelinating lesions in MOGAD are associated with the loss of Cx32 and Cx47 GJs.

Although some patients with leukodystrophy-like phenotypes demonstrate radiological features at onset, as observed in patient 1 in this report, some also have a lag between the presentation of clinical and radiological features, as seen in patient 2. This lagging could be attributed to the progressive loss of tissue integrity, leading to increased neuroaxonal injury, diffuse acquired white matter injury, and gradual cumulation over time. Although infrequent, the leukodystrophy-like phenotype of MOGAD is a treatable disorder compared with genetic leukodystrophies. Patients with extensive and confluent white matter lesions require early testing of MOG-IgG to ensure early administration of treatments.

In summary, our cases revealed an uncommon pattern of MOGAD with features of atypical clinical presentations and widespread changes in the white matter, overlapping with brain MRI findings of genetic or metabolic leukodystrophies. The leukodystrophy-like pattern of MOGAD may show a distinct progressive and recurrent phenotype, leading to a poor neurological prognosis. Recognizing that MOGAD treatment may need to be escalated beyond the current therapy in refractory cases is important. Further research is needed to determine whether such cases can be included in the MOGAD spectrum as a reference for diagnostic criteria and prognosis prediction. Moreover, research on pathobiological processes may provide new insights and improve the prognosis of this distinct group of patients.

## Data Availability

Not applicable.

## Abbreviations

ADS: Acquired demyelinating syndromes
MOG: Myelin oligodendrocyte glycoprotein
MOG-IgG: Myelin oligodendrocyte glycoprotein immunoglobulin
MOGAD: Myelin oligodendrocyte glycoprotein associated disorder
CSF: Cerebrospinal fluid
CNS: Central nervous system
MRI: Magnetic resonance imaging
ADEM: Acute disseminated encephalomyelitis
ON: Optic neuritis
NMOSD: Neuromyelitis optica spectrum disorders
EEG: Electroencephalogram
ANA: Antinuclear antibodies
ANCA: Antineutrophil cytoplasmic antibodies
NMDAR: Anti-N-methyl-D-aspartate receptor
IV: Intravenous
WISC: Wechsler Intelligence Scale for Children
IQ: Intelligence quotient
GJ: Gap junction
Cx47: Connexin47
Cx32: Connexin32
